# Analysis of Medications Returned During a Medication Take-Back Event

**DOI:** 10.3390/pharmacy3030079

**Published:** 2015-07-27

**Authors:** Christina H.J. Yang, Mitesh Doshi, Nancy A. Mason

**Affiliations:** 1 Nash Drugs, Inc., Hillsdale, MI 49242, USA; 2 Beaumont Health System, Royal Oak, MI 48073, USA; E-Mail: Mitesh.Doshi@beaumont.edu; 3 University of Michigan College of Pharmacy, 428 Church Street, Ann Arbor, MI 48109, USA; E-Mail: nmason@med.umich.edu

**Keywords:** adherence, medication safety, unused medication, medication take-back, medication disposal

## Abstract

A medication take-back event was held in Lansing, MI, USA, for four hours in September 2013. The objective was to quantify medication waste by determining the ratio of medication units remaining *versus* dispensed and to identify therapeutic classes with greater ratios of remaining medication units. Drug name, strength, quantity remaining, quantity dispensed, dispensary source, and brand or generic were recorded from the label of each medication container returned. Out of the over 3600 medication containers collected, this study analyzed 2459 containers, which included 304 controlled substances. On average, 66 percent of the medications dispensed in these containers were unused, and therefore wasted. Immunologic medications had the lowest quantity of waste at 54%, while geriatrics/miscellaneous therapeutic class yielded the highest quantity of waste at 79%. The most common therapeutic classes collected were pain/spasm, cardiovascular, and mental health. Greater emphasis on patient education regarding medication adherence and health care professionals’ judicious prescribing habits is warranted to reduce the frequency of unused medications. The increased accessibility to medication return sites may alleviate the prevalence of medication accumulation, environmental damage, and medication misuse.

## 1. Introduction

Unused medications may accumulate in home medicine cabinets due to a variety of reasons including overprescribing, elimination of need for the medication, noncompliance to the regimen, or medication expiration. People with access to medicine cabinets can divert medications for use by someone other than the patient or use the medications for unintended purposes. Medications may also be disposed into the trash, toilet or sink, risking the safety of others and damaging the environment. Even when there is no intention for mishandling, patients may retain medications due to the high cost of the medication, lack of disposal method, or the possibility of needing these medications again in the future.

Accessible storage of these medications poses a particular threat to children and elders, who may not understand the dangers of medications and may inadvertently ingest these medications, with consequences ranging from stomachache to death. In 2011, more than 68,000 children in the US suffered from medication poisoning and were seen in emergency departments, with 86% of these cases involving children ingesting adult medicine [1]. The most common places children found medications included on the ground, in purses or bags, on counters or nightstands, in pillboxes, and in cabinets or drawers [1]. Out of the more than two million calls made annually to poison control centers regarding unintentional child poisonings, nine out of 10 involve poisonings at home [2].

More common than accidental ingestion is the misuse of medications by young adults and adolescents with 21.7% and 13.0% of 12th graders in the US admitting to medical misuse of prescription medications and narcotics, respectively, as surveyed by the National Institute of Drug Abuse in 2014 [3]. Nationally, 4.5 million people over the age of 11 reported being current nonmedical users of prescription pain relievers, tranquilizers, stimulants, and sedatives based on the 2013 National Survey on Drug Use and Health [4]. Fifty-three percent of these nonmedical users stated they received analgesics for free from a friend or relative [4]. The reported 6.5 million Americans abusing prescription drugs in 2013 is more than double the number of those who have admitted to using heroin, cocaine, and hallucinogens combined [4]. With this high prevalence of medication being used for unintended persons or purposes, it can be deduced that, for many patients, the quantity of medications prescribed must far exceed the quantity needed for intended treatment.

The public media and scientific community have also highlighted the environmental impact of disposed medications. Flushing medications down the toilet or sink, disposal in household trash, and normal human and animal excretions containing pharmaceutical ingredients can all lead to medications being released into the environment [5]. On a list of more than 100 active ingredients found in the environment, amoxicillin, acetaminophen, and metoprolol stand at the top [6]. Unfortunately, some of these drugs that have seeped into the sewage system have failed to be eliminated through conventional sewage treatment, rendering the public and the environment more susceptible to contaminated water and antibiotic resistance [6,7]. Studies examining the effects of medication-contaminated drinking water have not yet produced reliable results. Additionally, there is a lack of information regarding biodegradation of these chemicals in soil environments, further providing concern for public and environmental safety.

To help relieve these concerns, the Drug Enforcement Administration (DEA) sponsored a National Prescription Drug Take-Back Day biannually where the public can bring their unused medications to designated sites. This program collected 309 tons of unwanted medications at 5495 locations during the most recent Drug Take-Back Day in September 2014, with each year’s collections reported greater than the previous year’s [8]. While this event cleared out medicine cabinets of many patients across the country, the increasing utilization of these types of events shows that there are still homes with unused or expired medications and an ongoing accumulation through new prescriptions. In response to the Secure and Responsible Drug Disposal Act, enacted by Congress in 2010, the DEA implemented new disposal regulations that allow retail pharmacies to register to become authorized collectors of controlled medications, replacing the biannual take-back events [8].

Because of the growing concern of accumulating unused medications, this study was initiated with the purpose to assess the quantity of medications gathering in homes by determining the ratio of medication units remaining *versus* dispensed, and to identify the therapeutic classes with greater ratios of remaining medication units.

## 2. Methods

A medication take-back event, held for four hours on 10 September 2013, was the site for data collection. The event was part of the Michigan Pharmacists Association’s (MPA) annual Pharmacy Day at the Capitol. Event volunteers consisted of student pharmacists and faculty of Ferris State University, Wayne State University, and the University of Michigan attending Pharmacy Day at the Capitol, as well as MPA staff. Because this operation included controlled medications, local police officers supervised all aspects of the event from medication collection to disposal and incineration.

All members of the public were welcome to drop-off their unused medications. These medications were then counted and the following information was recorded and transcribed onto Microsoft Excel spreadsheets: drug name, strength, amount remaining, amount prescribed, generic or brand, and source (local pharmacy, mail-order pharmacy, or sample). Collected medications were transported from the site for incineration upon the conclusion of the event.

In addition to collecting medications, event volunteers asked all donors to complete an optional nine-question anonymous web-based survey (Appendix) within the following week to further gather information about their medication disposal habits. The survey was not administered on-site to allow for donors to quickly donate their medications and to minimize the wait time to do so.

Statistical analysis was completed only for prescription medications in tablet or capsule formulations. Medications were categorized into the following therapeutic classes: allergy (antihistamines), cardiovascular, dietary (related to electrolyte or vitamin imbalances), endocrine (e.g., diabetes, hypothyroidism, reproductive hormone replacement), gastrointestinal, immunologic (e.g., cancer, rheumatoid arthritis, immunosuppression), infectious disease, mental health (e.g., depression, bipolar disorder, schizophrenia, anxiety, attention deficit disorder, insomnia, seizure disorder), nausea, pain/spasm, respiratory, and geriatric/miscellaneous (e.g., urinary incontinence, osteoporosis, dementia, Parkinson’s disease, benign prostatic hyperplasia, erectile dysfunction). Exclusion criteria included medications never available in the US, pet medications, medications in containers without a legible label, and containers with remaining medication amounts larger than the amount dispensed as this may have been the result of patients combining medications into the same container. Medications not in tablet or capsule formulations were also excluded as limited package sizes may intend for patients to have more than needed. For example, amoxicillin suspension is manufactured in 100 and 150 mL formulations, but a patient may require only 120 mL of the 150 mL formulation to complete therapy.

Medications analyzed in this study were grouped by therapeutic classes except the grouping called geriatric/miscellaneous. Medications were quantified by the average ratio of the quantity of units remaining out of the quantity dispensed. The averages were compared using a one-way analysis of variance (ANOVA), and a two-sample unpaired Student’s *t*-test with Bonferonni adjustment evaluated the statistical difference between the averages of each therapeutic class to each other. A *p*-value less than 0.05 was considered statistically significant. To avoid confusion, the term “container” will be used to describe the box, bottle, or other packaging the medications were enclosed in.

## 3. Results

The event gathered 3633 containers of medications, including over-the-counter, prescription, and pet medications. Out of 2,830,094 units (tablets or capsules) originally dispensed in these containers, 1,824,854 units of medications were collected. The top 15 most commonly returned out of the 348 different prescription drugs collected are listed in Table 1. The majority of the medications were generic products, composing 69.0% of all medications collected. Most were obtained at local pharmacies (89.4%), compared to mail order (5.7%), or samples from physician offices (4.8%).

**Table 1:** Quantities of most commonly returned medications.

	Medication	Quantity of Containers	Percentage of All Containers Returned
1	Acetaminophen/hydrocodone	110	4.4
2	Ibuprofen	73	2.9
3	Tramadol	44	1.8
4	Acetaminophen/codeine	42	1.7
5	Naproxen	42	1.7
6	Prednisone	41	1.7
7	Amoxicillin	37	1.5
8	Omeprazole	37	1.5
9	Levothyroxine	35	1.4
10	Aspirin	32	1.3
11	Acetaminophen/propoxyphene	27	1.1
12	Cyclobenzaprine	27	1.1
13	Cephalexin	26	1.1
14	Lisinopril	26	1.1
15	Warfarin	26	1.1

**Figure 1:**
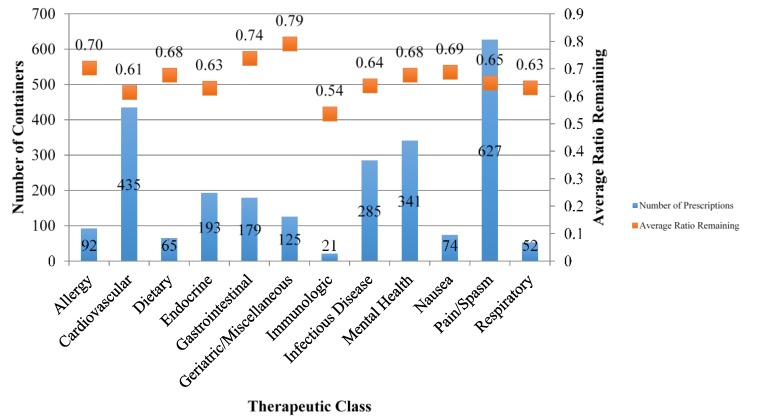
Quantities of containers collected and average ratios remaining categorized by therapeutic class.

For this study 2459 containers were analyzed, with 304 of them holding controlled medications (excluding tramadol as it was not a controlled substance in the state of Michigan at the time of the event). Figure 1 displays the quantity of containers collected at the event and the respective average ratio of remaining units of medications per container, grouped by therapeutic drug class. The pain/spasm therapeutic class accounted for the largest number of containers collected with 615 containers, followed by 431 containers of cardiovascular medications, and 341 containers of mental health medications.

The overall ratio of remaining medication units to units dispensed was 0.66 for all medications included in this study. A one-way ANOVA was calculated on the average ratios, grouped by therapeutic class. The analysis revealed a significant difference among the therapeutic groups’ ratios of remaining units, F(11, 2447) = 3.37, *p* < 0.001. The average ratio remaining for each therapeutic drug class was compared to that of the other therapeutic classes utilizing Student’s *t*-tests with Bonferonni adjustment. Six pairs showed a significant difference, five of which included the geriatric/miscellaneous therapeutic class: geriatric/miscellaneous > cardiovascular (*p* < 0.001), geriatric/miscellaneous > endocrine (*p* < 0.001), geriatric/miscellaneous > infectious diseases (*p* < 0.001), geriatric/miscellaneous > mental health (*p* = 0.027), geriatric/miscellaneous > pain (*p* < 0.001), and gastrointestinal > cardiovascular (*p* = 0.001).

**Figure 2:**
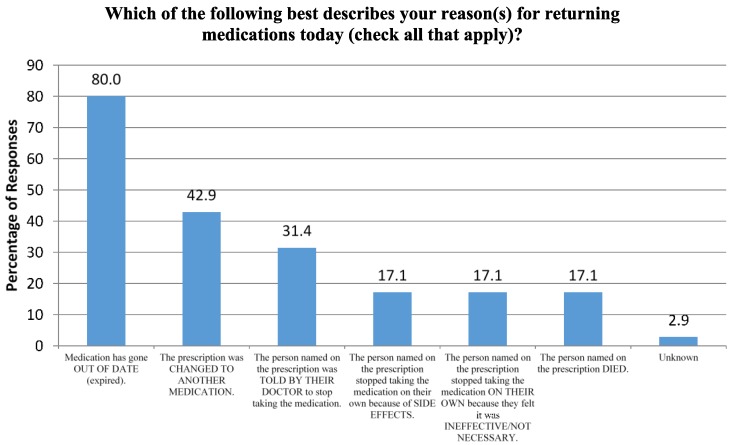
Participant Survey Question.

Seventy participants responded to the online survey. Fifty of the seventy respondents were female, and nearly 60% were adults above the age of 50. Over 75% of respondents reported returning a family member’s medication(s), and 67% reported returning their own. Medication expiration was the most common reason for medication disposal at the event, while discontinuation of the medications due to side effects or lack of necessity without professional recommendation were the least common reasons (Figure 2). Nearly 59% of respondents cited drug take-back events as their usual method for disposing unused medications, while over 25% stated they disposed of them in the garbage and 14% flushed medications down the toilet or sink.

## 4. Discussion

While this event gathered 345 different prescription medications, the top five most common were pain relievers. The pain/spasm therapeutic class had the greatest number of containers collected, with more than a quarter of all containers analyzed falling into this category. Likewise, similar medication take-back events in rural Appalachia and Findlay, Ohio gathered high rates of pain medications, with hydrocodone-, propoxyphene-, and codeine-containing medications being three of the most common medications collected [9,10]. A survey of Cook County, Illinois, residents by researchers at the University of Illinois revealed the first and the fifth most common type of medication in homes were over-the-counter pain (82.5% of respondents) and prescription pain (39.2%) medications, respectively [11]. The similarity of these studies justifies the widespread concern that patients are accumulating analgesics in the home, which provides opportunity for abuse, diversion, and accidental ingestion.

The high rate of having unused pain relievers in the home not only raises the concern of potential misuse, but the concern that pain relievers may be overprescribed. Thirty-seven percent of the Cook County survey participants stated they had leftover unexpired medications from a previous illness [11]. The most common reasons for this were that they felt better or the directions stated take as needed and the drug was no longer necessary. As patients prescribed analgesics for short-term use are directed to take as needed, a portion of the quantity returned during the event was due to prescribing a greater amount of analgesics than the patients found necessary.

Across all therapeutic categories, the proportion of remaining medications was fairly consistent, with nine of the 12 categories falling between the range of 60% to 70%. This narrow range also yields great concern related to patient nonadherence. The data show remaining proportions are similar despite differences in acute or chronic use, such as between medications for nausea (0.69) *vs.* endocrine disorders (0.63).

While the medications included in the geriatric/miscellaneous class are not exclusive to the elderly population, this class interestingly had a relatively high ratio of remaining medications and was included in five of the six comparison groups that noted a statistically significant difference in average ratios. The high use of these medications by the elderly population may be the result of having greater time to accumulate unused medications, potentially due to dose adjustments or therapy modifications, or to caretakers or family members disposing of medications after the patient’s death.

Eighty percent of survey respondents cited medication expiry as a reason for disposing medications. This high number may be because the survey allowed for donors to indicate multiple reasons. For example, a patient may have selected two answers if he was told to discontinue a medication by his physician and kept it at home for several years before disposing of it at this event. The fact that the majority of respondents cited drug take-back events as their usual method for medication disposal is alarming because these events are only offered to local residents a few days of the year, if any, for some areas of the country. As medication accumulation is ongoing, the DEA’s intention of allowing pharmacies to register to take back controlled and noncontrolled medications would allow avenues for greater access to environmentally safe medication disposal.

There were several limitations in this study. Numerous reasons aside from non-adherence and overprescribing contribute to medications being unused. Patient allergies or intolerances, ineffective therapeutic effect, or diagnosis changes may have restricted patients from taking the full amount of medication dispensed. Data gathered during a medication take-back day are only reflective of a small percentage of the population in Lansing, MI and the surrounding areas who willingly disposed of their unused medications. The participants were primarily older adults, which is expected as this demographic tends to have more diseases and disorders for which medications are needed. The total number of participants was not recorded due to the operational logistics of this medication take-back event, but the number of survey respondents reflects a small percentage of this total.

## 5. Conclusion

Among all medications collected at the medication take-back event, approximately two-thirds of the quantity dispensed was remaining. Only 17% of survey respondents admit to discontinuing use on their own accord as they felt the medication was unnecessary, while the most common reason for donating medications to the event was medication expiration.

Currently, there are limited works published regarding what medications are collected at take-back events. Our study broadly matches the data gathered by groups in Findlay, Ohio, and rural Appalachia, with the high quantities of analgesics and cardiovascular agents collected during the event [9,10]. However, more studies are needed to further assess the incidence of drug accumulation and patients’ reasons for disposal.

The popularity of medication take-back events has primarily focused on disposing of unused medications rather than addressing the sources of this accumulation, namely overprescribing and the patient’s failure to complete full courses of therapy. While health care professionals should continue to stress the importance of patient adherence, prescribers can help reduce medication accumulation by prescribing more conservative quantities for medications. Local pharmacies can also aid in reducing accumulation by registering as authorized medication return sites with the DEA, which would offer patients an environmentally friendly method of medication disposal nearly every day of the year.
